# Biological Activities of Phosphocitrate: A Potential Meniscal Protective Agent

**DOI:** 10.1155/2013/726581

**Published:** 2013-07-11

**Authors:** Yubo Sun, Andrea Roberts, David R. Mauerhan, Andrew R. Sun, H. James Norton, Edward N. Hanley

**Affiliations:** ^1^Department of Orthopedic Research Biology, Cannon Research, Carolinas Medical Center, P.O. Box 32861, Charlotte, NC 28232, USA; ^2^Dixon Advanced Analytics Group, Carolinas Medical Center, P.O. Box 32861, Charlotte, NC 28232, USA

## Abstract

Phosphocitrate (PC) inhibited meniscal calcification and the development of calcium crystal-associated osteoarthritis (OA) in Hartley guinea pigs. However, the mechanisms remain elusive. This study sought to examine the biological activities of PC in the absence of calcium crystals and test the hypothesis that PC is potentially a meniscal protective agent. We found that PC downregulated the expression of many genes classified in cell proliferation, ossification, prostaglandin metabolic process, and wound healing, including bloom syndrome RecQ helicase-like, cell division cycle 7 homolog, cell division cycle 25 homolog C, ankylosis progressive homolog, prostaglandin-endoperoxide synthases-1/cyclooxygenase-1, and plasminogen activator urokinase receptor. In contrast, PC stimulated the expression of many genes classified in fibroblast growth factor receptor signaling pathway, collagen fibril organization, and extracellular structure organization, including fibroblast growth factor 7, collagen type I, alpha 1, and collagen type XI, alpha 1. Consistent with its effect on the expression of genes classified in cell proliferation, collagen fibril organization, and ossification, PC inhibited the proliferation of OA meniscal cells and meniscal cell-mediated calcification while stimulating the production of collagens. These findings indicate that PC is potentially a meniscal-protective agent and a disease-modifying drug for arthritis associated with severe meniscal degeneration.

## 1. Introduction

Osteoarthritis (OA) is one of the most prevalent causes of disability in the aging population and has enormous economic and social consequences. However, existing nonsurgical treatment options only provide symptomatic relief but have no effect on the progression of the underlying disease or cartilage degeneration. The lack of progress in the development of disease-modifying drugs for OA therapy is largely due to our limited understanding of the pathogenesis of OA and our insufficient knowledge about the molecular targets for OA therapy.

Knee OA is not merely an articular cartilage disease, but a disease of the whole joint. An important local factor is the structural integrity of the menisci. In recent years, there has been a dramatic advance in our understanding of the integral role of the menisci for knee function and the consequences of meniscal abnormality on the development of OA. Studies have found that meniscal degeneration is a general feature of knee OA and contributes to joint space narrowing [1, 2]. Meniscal lesions at baseline were more common in knees that developed OA than in the knees that did not develop OA during a 30-month follow-up period [3]. OA meniscal cells displayed a distinct gene expression profile different from normal meniscal cells [4]. These observations indicate that the meniscus is not a passive bystander in the disease process of knee OA [5, 6].

Basic calcium phosphate crystal and calcium pyrophosphate dihydrate crystal are the two most common articular calcium crystals. The presence of these crystals in OA articular cartilage and synovial fluid is well recognized. These crystals are also present in knee menisci of patients with end-stage OA [7, 8]. Studies found that these crystals stimulate cell mitogenesis, cell endocytotic activity, and the production of matrix metalloproteinases (MMPs) and inflammatory cytokines including interleukin-1 (IL-1) and prostaglandin-endoperoxide synthase 2/cyclooxygenase-2 (PTGS2/Cox-2) [9–11]. However, there is still controversy as to whether these crystals are causative factors, factors that exacerbate OA, or simply bystanders.

Phosphocitrate (PC) is a naturally occurring compound originally identified in rat liver mitochondrial extracts and crab hepatopancreas [12]. Moro et al. and Romanello et al. suggested that PC could be formed *in vivo* through cytosolic phosphorylation of citric acid [13, 14], which explains why it is nontoxic. Since its original identification, PC has been shown to be a powerful calcification inhibitor [15, 16]. Tew et al. speculated that PC prevented CaPO_4_ precipitation in cells or cellular compartments containing high concentration of Ca^2+^ and PO_4_ [12]. PC prevented soft tissue calcification *in vivo* and did not produce any significant toxic side effect in rats when administered through intraperitoneal injections in doses up to 150 *μ*mol/kg/day [17]. In cell cultures, PC inhibits calcium crystal-induced mitogenesis, expression of MMPs, and crystal-induced cell death [18–20]. In the Hartley guinea pig model of crystal-associated OA, PC inhibited meniscal calcification and reduced the severity of cartilage degeneration [21]. These observations provide support for the notion that crystals may play an important role in the development of OA and that calcification inhibitors are potentially disease-modifying drugs for crystal-associated OA therapy. However, two bisphosphonates, which are potent calcification inhibitors, failed to inhibit the development of OA in animal models of OA [22, 23], raising doubts as to whether calcification inhibitors are disease-modifying drugs for crystal-associated OA as well as the exact role of calcium crystals in the development of OA. In this study, we sought to examine the biological activities of PC in the absence of calcium crystals and test the hypothesis that PC has unique crystal-independent biological activities which may be responsible, at least in part, for its disease-modifying activity on OA and that PC is potentially a meniscal protective agent. 

## 2. Materials and Methods

Dulbecco's Modified Eagle Medium, StemPro chondrogenesis differentiation medium, fetal bovine serum, Hank's balanced salt solution, and stock antibiotic and antimycotic mixture were products of Invitrogen (Carlsbad, CA, USA). PC was synthesized according to the procedure described [24]. All other chemicals are purchased from Sigma (St. Louis, MO, USA). 

### 2.1. Cells

OA meniscal cells were prepared from menisci derived from patients with end-stage OA. Briefly, the medial menisci derived from patients with end-stage OA were processed to remove fatty and synovial tissues, minced into small pieces, and cultured in 100 mm plates at 37°C in medium containing 0.5% antibiotic/antimycotic solution and 10% serum. Every three or four days, the culture medium was changed. When the cells reached 70% confluence, they were passaged and maintained in medium containing 10% serum. Human foreskin fibroblasts were obtained from American Type Culture Collection (CRL-2429, Manassas, VA, USA). OA meniscal cells prepared from three OA patients were used in this study. OA menisci were collected with the approval of the authors' Institutional Review Board from OA patients undergoing knee joint replacement surgery. The need for informed consent was waived because those menisci were surgical waste, and no private patient information was collected. 

### 2.2. Cell Culture and RNA Extraction

OA meniscal cells derived from three OA patients were harvested from cell culture plates and mixed and replated in four 100 mm cell culture plates at 90% confluence. On the second day, medium containing 1% serum was added. Twenty-four hours later, the medium in two plates was replaced with medium containing 1% serum and PC (1 mM), and the medium in the other two plates was replaced with medium containing 1% serum without PC. Twenty-four hours later, total RNA was extracted from these cells using TRIzol reagent (Invitrogen, Carlsbad, CA, USA) and purified using Oligotex kit (Qiagen, Valencia, CA, USA). We repeated the experiment twice. Microarray was performed using these RNA samples (total six RNA samples). 

### 2.3. Microarray

RNA samples extracted from three independent experiments were used for microarray analysis experiments. Briefly, double-stranded DNA was synthesized using SuperScript Double-Stranded cDNA Synthesis Kit (Invitrogen, San Diego, CA, USA). The DNA product was purified using GeneChip Sample Cleanup Module (Affymetrix, Santa Clara, CA, USA). cRNA was synthesized and biotin-labeled using BioArray high yield RNA transcript labeling kit (Enzo Life Sciences, Farmingdale, NY, USA). The cRNA product was purified using GeneChip Sample Cleanup Module and subsequently chemically fragmented. The fragmented and biotinylated cRNA was hybridized to HG-U133_Plus_2 GeneChip using Affymetrix Fluidics Station 400 (Affymetrix, Santa Clara, CA, USA). The fluorescent signals were quantified during two scans by Agilent Gene Array Scanner G2500A (Agilent Technologies, Palo Alto, CA, USA) and GeneChip operating Software (Affymetrix, Santa Clara, CA, USA). Genesifter (VizX Labs, Seattle, WA, USA) was used for the analysis of differential gene expression and gene ontology. 

### 2.4. Real-Time RT-PCR

After microarray analyses, we mixed the RNA samples extracted from PC-treated OA meniscal cells (PC-treated RNA sample) and the RNA samples extracted from untreated OA meniscal cells (untreated RNA sample) and performed RT-PCR experiments. Briefly, cDNA was synthesized using TaqMan Reverse Transcription Reagents (Applied Biosystems, University Park, IL, USA) using the RNA samples described. Quantification of relative transcript levels for selected genes and the housekeeping gene glyceraldehyde 3-phosphate dehydrogenase (GAPDH) was performed using ABI7000 Real-Time PCR system (Applied Biosystems, University Park, IL, USA). TaqMan Gene Expression assay (Applied Biosystems, University Park, IL, USA) was used. CDNA samples were amplified with an initial Taq DNA polymerase activation step at 95°C for 10 minutes, followed by 40 cycles of denaturation at 95°C for 15 seconds and annealing at 60°C for one minute. Fold change was calculated, and the expression level of the genes to be examined was normalized to the expression level of GAPDH. RT-PCR experiment was performed in triplicates using the same RNA sample.

### 2.5. Cell Proliferation

OA meniscal cells (2 × 10^4^) were plated in six well cluster plates and cultured in medium containing 10% serum in the presence of increasing amounts of PC (triplicates). Medium was changed every three days until the OA meniscal cells in the wells without PC reached 85% confluence. Cells were then harvested and cell numbers were determined using a hemocytometer. This experiment was repeated 3 times using OA meniscal cells derived from different patients. This proliferation assay was also performed using human primary foreskin fibroblasts.

### 2.6. Micromass Culture and Histology

OA meniscal cells were harvested from several 100 mm culture plates and suspended in medium containing 10% serum. For preparing a micromass, a droplet of the cell suspension containing 6 × 10^6^ cells was placed in a well of a 24-well plate. After placing all droplets, the plate was incubated for 4 hours at 37°C in a tissue culture incubator. These micromasses were then fed with StemPro chondrogenesis differentiation medium with PC (1 mM) or without PC every three days throughout the experiment for 14 days. Each well was then rinsed twice with 500 *μ*L of Hank's balanced salt solution, and two drops of eosin were added to these wells. Five minutes later, eosin was aspirated off and the micromasses were transferred to a strip of filter paper which sat on the top of an ethanol-soaked sponge within a plastic cassette. The cassettes sat in a 10% formalin solution for one hour. These micromasses then underwent routine paraffin embedding. Sections were cut at 5 *μ*m thick and stained with picrosirius red for collagens, alcian blue for proteoglycans, and alizarin red for calcium deposits. 

### 2.7. Cell-Mediated Calcification in Monolayer Culture

OA meniscal cells were plated in twenty-four well plates at 90% confluence. The next day, medium was replaced with StemPro chondrogenesis differentiation medium containing 1 mM adenosine-5′-triphosphate (ATP) in the absence or presence of PC (1 mM). The cells were cultured for 14 days and fed with StemPro chondrogenesis differentiation medium containing ATP every three or four days throughout the experiment. At the end of the experimental period, media were removed. Calcification was examined using alizarin red.

### 2.8. Statistical Analysis

For cell proliferation, data are expressed as the mean ± SD, and the difference between two groups was analyzed using Student's *t*-test. For real-time RT-PCR, experiment was repeated in triplicates. The difference between two experimental groups was analyzed using Student's *t* test. In all cases, *P* values less than 0.01 were considered significant. Statistical analysis was performed using the SAS software, version 9.3.

## 3. Results

### 3.1. Effect of PC on Gene Expression

We performed three microarray experiments (PC-treated RNA sample I and untreated RNA sample I; PC-treated RNA sample II and untreated RNA sample II; PC-treated RNA sample III and untreated RNA sample III) as described. The results of the three microarray experiments were quite similar. The results of the first microarray experiment showed that of the more than 50,000 transcripts, 2445 transcripts displayed significant differential expression (more than 1.6-fold changes) in the PC-treated OA meniscal cells compared with the untreated OA meniscal cells. A total of 1795 transcripts displayed decreased expression and 650 transcripts displayed increased expression. The genes that fell into specific biological processes previously implicated in OA, or suspected to have a role in OA are listed in Tables 1 and 2.

As shown in Table 1, the expression of numerous genes classified in cell proliferation was downregulated by PC. Of the 62 differentially expressed genes (more than 2-fold changes) classified in cell proliferation, the expression of 52 genes, including bloom syndrome recq helicase-like (BLM; −7.41-fold change), lymphoid-specific helicase (Hells; −4.46-fold change), cell division cycle 7 homolog (CDC7; −4.11-fold change), CDC 25 homolog C (CDC25C; −2.36-fold change), and cyclin E2 (CCNE2, −3.51-fold change), was downregulated by PC. This specific downregulatory effect of PC on the expression of genes classified in cell proliferation suggests that PC may inhibit the proliferation of OA meniscal cells.

The expressions of many genes classified in ossification and bone morphogenetic protein (BMP) signaling pathway were also downregulated by PC (Table 1). Of the 22 differentially expressed genes classified in ossification, the expression of 13 genes, including collagen, type XIII, alpha 1 (COL13A1; −3.39-fold change), special AT-rich sequence-binding protein (SATB2; −3.13), mothers against DPP homolog 1/SMAD family member 1 (SMAD1; −2.19), ectonucleotide pyrophosphatase/phosphodiesterase 1 (ENPP1; −2.02-fold change), and ankylosis progressive homolog (ANKH; −1.60-fold change), was downregulated by PC. The expression of 9 genes, including insulin-like growth factor-binding protein 5 (IGFBP5; 1.78-fold change) and matrix Gla protein (MGP; 1.61-fold change), was upregulated by PC. Of the 8 differentially expressed genes classified in BMP signaling pathway, the expression of all 8 genes, including BMP receptor, type IB (BMPR1B; −1.93-fold change), was downregulated by PC. 

In addition, the expression of several genes classified in prostaglandin metabolic process and respond to wounding was downregulated by PC (Table 1). Of the 5 differentially expressed genes classified in prostaglandin metabolic process, the expression of 3 genes, including prostaglandin-endoperoxide synthase 1/cyclooxygenase-1 (PTGS1/Cox-1; −2.13-fold change), phospholipase A2, group IVA (PLA2G4A; −1.61-fold change), was downregulated by PC. Of the 9 differentially expressed genes classified in respond to wounding, the expression of 7 genes, including thrombomodulin (THBD; −4.24-fold change), plasminogen activator, tissue (PLAT, −3.12-fold change) and plasminogen activator, urokinase receptor (PLAUR; −1.94-fold change), was downregulated by PC.

Many genes that were upregulated by PC fell into the biological processes of fibroblast growth factor (FGF) receptor signaling pathway, collagen fibril organization, and extracellular structure organization (Table 2). Of the 7 differentially expressed genes classified in FGF receptor signaling pathway, the expression of 5 genes, including FGF7 (1.83-fold change), FGF9 (2.38-fold change), and FGF receptor 2 (FGFR2; 2.20 fold change), was upregulated by PC. Of the 6 differentially expressed genes classified in collagen fibril organization, the expression of all 6 genes, including collagen type I, alpha 1 (COL1A1; 1.62-fold change), collagen type XI, alpha 1 (COL11A1; 1.82-fold change), and collagen type XIV, alpha 1 (COL14A1; 2.06-fold change), was upregulated by PC. Further examination of the microarray data indicated that the expression of collagen type V, alpha 1 (COL5A1; 1.52-fold change) and collagen type V, alpha 2 (COL5A2; 1.44-fold change) was also upregulated by PC although the changes were less than 1.6-fold (Table 2). Of the 15 genes differentially expressed genes classified in extracellular structure organization, the expression of 13-genes, including extracellular matrix protein 2 (ECM2; 2.04-fold change), was upregulated by PC. Only 2 genes, including MMP9 (−1.91-fold change), were downregulated by PC (Table 2). 

Finally, of the 26 genes classified in inflammatory response, the expression of 15 genes, including adenosine A1 receptor (ADORA1; 2.48-fold change), CD24 molecule (CD24; 2.09-fold change), and B-cell CLL/lymphoma 6 (BCL6; 1.98-fold change), was upregulated by PC. The expression of 11 genes, including NF-kappaB inhibitor zeta (NFKBIZ; −2.58-fold change), adrenergic, beta-2, receptor (ADRB2; −2.41-fold change), and chemokine (C-X-C motif) ligand 6 (CXCL6; −2.19-fold change), was downregulated by PC (Table 2).

### 3.2. Real-Time RT-PCR

Real-time RT-PCR was used to confirm expression of selected genes. The results are listed in Table 3. As shown, the differential expression of the genes examined was confirmed by real-time RT-PCR (*P* < 0.01).

### 3.3. PC Inhibited Proliferation of OA Meniscal Cells

**Figure 1:**
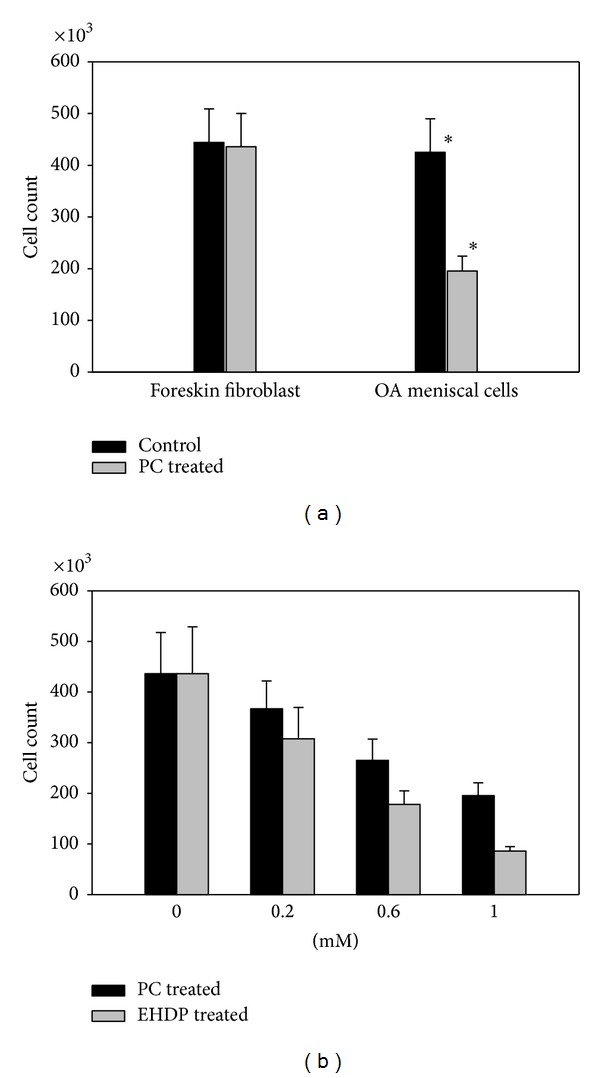
PC inhibited the proliferation of OA meniscal cells. OA meniscal cells and human foreskin fibroblast were plated in six-well cluster plates and cultured in the presence or absence of PC. (a) There were about 55% fewer OA meniscal cells in the PC-treated (1 mM PC) wells than that in the untreated wells (the right bar group; *P* = 0.0032). In contract, PC had no effect on the proliferation of human foreskin fibroblast (the left group bars). (b) PC and EHDP inhibited the proliferation of OA meniscal cells in a dose-dependent manner.

The specific downregulatory effect of PC on the expression of genes classified in cell proliferation suggests that PC may inhibit the proliferation of OA meniscal cells. To test this, we cultured OA meniscal cells in the absence and presence of PC for 9 days and then determined the cell number using a hemocytometer. Indeed, we found that PC inhibited the proliferation of OA meniscal cells (Figure 1(a), right bar group). There were 55% fewer OA meniscal cells in the PC-treated wells than that in the untreated wells (*P* < 0.01). In contrast, PC had no effect on the proliferation of human foreskin fibroblasts (Figure 1(a), left bar group). For comparison, we also examined the effect of disodium salt of ethane-1-hydroxy-1, 1-bisphosphonic acid (EHDP), which is a bisphosphonate, together with PC on the proliferation of OA meniscal cells. As shown in Figure 1(b), both PC and EHDP inhibited the proliferation of OA meniscal cells in a dose-dependent manner. In addition, the morphology of PC-treated OA meniscal cells and untreated OA meniscal cells was similar (not shown), indicating that the reduction in cell number was not due to cellular toxicity of PC.

### 3.4. PC Stimulated Production of Collagens

**Figure 2:**
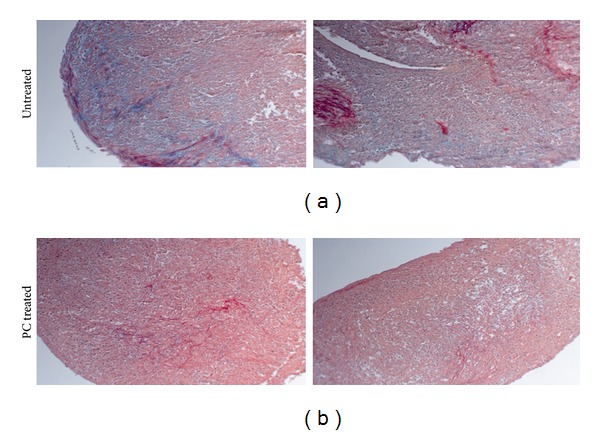
PC stimulated the production of collagens. Micromasses of OA meniscal cells were cultured in the absence of PC ((a); magnification 10x) or the presence of 1 mM PC ((b); magnification 10x) for 14 days. These micromasses were then processed and stained with picrosirius red. Note the much stronger picrosirius red staining in the PC-treated micromass than that in the untreated micromass.

The upregulatory effect of PC on the expression of genes classified in collagen fibril organization, including COL1A1 (1.62-fold change), COL11A1 (1.82-fold change), COL14A1 (2.06-fold change), COL5A1 (1.52-fold change), and COL5A2 (1.44-fold change), suggests that PC may stimulate the production of collagens by the OA meniscal cells. To examine this, we prepared micromasses of OA meniscal cells (triplicates) and examined the production of collagens using picrosirius red staining. The results confirmed that PC stimulated the production of collagens by OA meniscal cells. Representative images of picrosirius red staining are shown in Figure 2. As shown, the picrosirius red staining in the PC-treated micromasses (Figure 2(b)) was stronger than that in the untreated micromasses (Figure 2(a)). These micromasses were also stained with alcian blue. Results indicated that PC had no detectable effect of the production of proteoglycans (not shown).

### 3.5. PC Inhibited Meniscal Cell-Mediated Calcification

**Figure 3:**
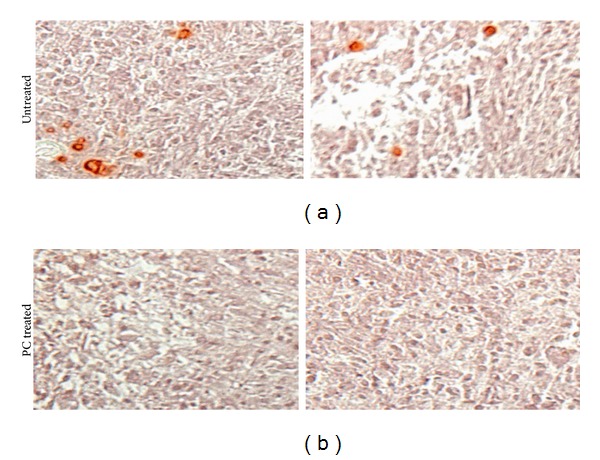
PC inhibited OA meniscal cells-mediated calcification in micromass culture. Micromasses of OA meniscal cells were stained with alizarin red. In the absence of PC, calcium deposits were detected ((a); magnification 10x). In the presence of 1 mM PC, calcium deposits were abolished ((b); magnification 10x).

These micromasses were also stained with alizarin red for calcium deposits. Representative images of alizarin red staining are shown in Figure 3. As shown, many calcium deposits were detected in the micromasses of OA meniscal cells cultured in the absence of PC (Figure 3(a)), but no calcium deposits were detected in the micromasses cultured in the presence of PC (Figure 3(b)). PC treatment (1 mM) abolished OA meniscal cell-mediated calcium deposition in micromass culture completely.

**Figure 4:**
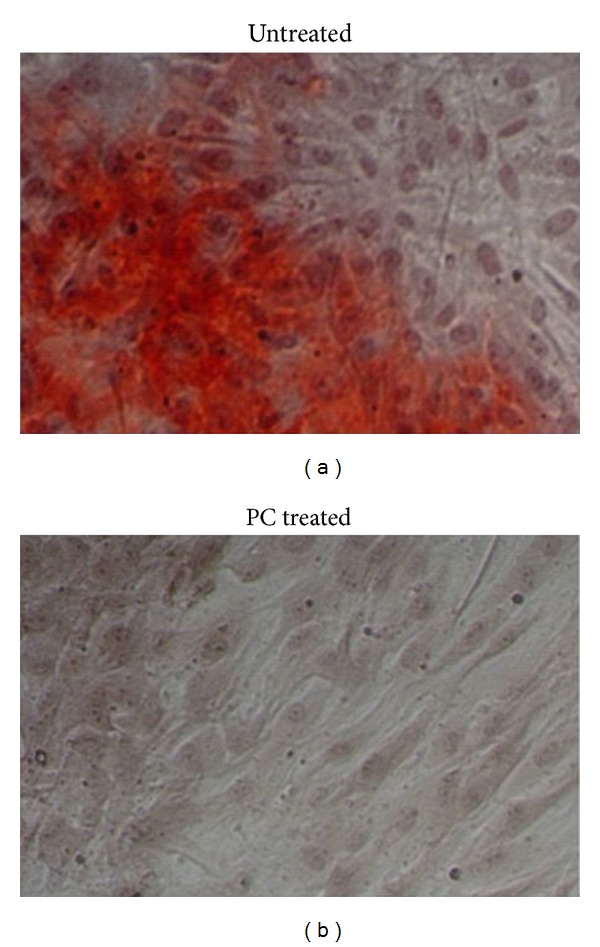
PC inhibited OA meniscal cells-mediated calcification in monolayer culture. In the absence of PC, calcium deposits were formed ((a); magnification 20x). In the presence of 1 mM PC, no calcium deposits were formed ((b); magnification 20x).

The inhibitory effect of PC on OA meniscal cell-mediated calcification was also examined using monolayer culture. As shown in Figure 4, calcium deposits were detected in the monolayer culture of OA meniscal cells (Figure 4(a), but no calcium deposits were detected in the monolayer culture of OA meniscal cells cultured in the presence of PC (Figure 4(b)). 

## 4. Discussion

Increased number and size of cell clusters are the hallmark histological feature of OA articular cartilage [25, 26]. Cell clusters are activated cells and represent an important source of pathological mediator. The number of proliferating chondrocytes increases during OA progression [26, 27]. Cell clusters are also detected in OA menisci [28]. These observations indicate that abnormal cell activation and proliferation may play a role in the development and/or progression of OA. In this study, we demonstrated that PC downregulated the expression of numerous genes associated with cell proliferation. Consistent with this regulatory effect, PC inhibited the proliferation of OA meniscal cells. Although the exact implication of this effect on the development of OA is unclear at present, these findings suggest that PC may inhibit the activation of OA meniscal cells or inhibit the formation of cell clusters. 

Studies consistently reported apoptotic cells in cell clusters [29, 30]. One of the mechanisms of apoptosis is abnormal hypertrophic differentiation of chondrocytes and subsequent pathological calcification. There is abundant evidence indicating the expression of hypertrophic makers, including collagen X, osteocalcin, and MMP13, within the cell clusters [31–33]. In addition, staining of OA cartilage sections showed colocalization of calcium deposits and clusters containing apoptotic cells adjacent to the calcium deposits [34]. Mineralization of extracellular matrix surrounding cell clusters was also detected in OA menisci [28]. These observations indicate that abnormal cell activation, terminal differentiation, pathological calcification, and apoptosis may be mutually linked disease processes in OA. In this study, we demonstrated that PC inhibited the expression of numerous genes associated with cell proliferation, differentiation, and pathological calcification, including CDC7 (−4.11-fold change), CDC25C (−2.36-fold change), COL13A1 (−3.39-fold change), SATB2 (−3.13-fold-change), SMAD1 (−2.19-fold change), ENPP1 (−2.02-fold change), and ANKH (−1.60-fold change). Consistent with this regulatory activity, PC inhibited the proliferation of OA meniscal cells and abolished meniscal cells-mediated calcification. These findings suggest that PC may exert its disease-modifying activity in part by targeting the genes associated with cell proliferation, differentiation, and pathological calcification. 

Several previous observations provide support for this potential mechanism. For example, studies found that COL13 was increased in OA articular cartilage and that transgenic mice overexpressing COL13 had abnormally high bone mineral density [35, 36]. The expression of ENPP1 and ANKH was elevated in OA meniscal cells, menisci, or articular cartilage [4, 34, 37]. SMAD1 induced terminal differentiation of chondrocytes and promoted calcification [38]. SATB2 stimulated osteogenic differentiation of adult stem cells [39]. These previous findings demonstrated that elevated expressions of COL13, ENPP1, ANKH, SMAD1, and SATB2 in OA cartilage or OA menisci were associated with terminal differentiation and biomineralization. The downregulatory effect of PC on these genes indicates that PC may reverse the processes of terminal differentiation and biomineralization. 

Early studies demonstrated that MGP was a calcification inhibitor and that inhibition of IGFBP5 breakdown reduced articular cartilage loss in an experimental OA [40, 41]. In this study, we demonstrated that PC upregulated the expression of MGP (1.61-fold change) and IGFBP5 (1.78-fold change). This upregulatory effect of PC may lead to elevated production of MGP and IGFBP5. 

FGF signaling pathway plays an important role in the regulation of osteogenesis and chondrogenesis. Studies found that FGF18 induced chondrocyte hypertrophy and mineralization [42, 43], while FGF9 inhibited terminal differentiation of calvaria-derived cells and mineralization [44, 45]. FGF7 was a potent inhibitor of phosphate transport [46]. In this study, we found that PC downregulated the expression of FGF18 (−1.73-fold change), while it upregulated the expression of FGF9 (2.38-fold change) and FGF7 (1.83-fold change). These regulatory effects indicate that PC may inhibit OA meniscal cell differentiation and pathological calcification in part by modulating the FGF signaling pathway. Consistently, studies have demonstrated that constitutive expression of thrombospondin 1 (THBS1), a protein involved in FGF signaling pathway, inhibited biomineralization and that THBS1 gene therapy suppressed the progression of arthritis in a rat model of OA [47, 48]. Another study demonstrated that the absence of signaling through FGF receptor 3 (FGFR3) leads to premature cartilage degeneration and early arthritis [49]. These previous findings indicate a key role of FGF signaling pathway in the development of healthy articular cartilage. The specific upregulatory activity of PC on THBS1 (1.68-fold change) and FGF9 (a preferred ligand for FGFR3) indicates that PC may exert its disease-modifying activity on OA in part by modulating the FGF signaling pathway.

Severe loss of collagen occurs in OA menisci [50]. Disease-modifying drugs targeting meniscal degeneration for OA therapy should inhibit meniscal collagen loss. In this study, we demonstrated that PC stimulated the production of collagen by OA meniscal cells. This finding suggests that PC is not only potentially a disease-modifying drug for OA therapy but also potentially a meniscal protective agent.

Nonsteroidal anti-inflammatory drugs (NSAIDs) are widely used to treat arthritis. Traditional NSAIDs inhibit both PTGS1/Cox-1 and PTGS2/Cox-2, while new generation of NSAIDs selectively inhibits PTGS2/Cox-2. In this study, we demonstrated that PC downregulated the expression of PTGS1/Cox-1 (Table 1, −2.13-fold change). Further examination indicated that PC also downregulated the expression of PTGS2/Cox-2 (Table 1, −1.33-fold change). Moreover, PC downregulated the expression of PLA2G4A (−1.61-fold change), PLAUR (−1.94-fold change), and MMP9 (−1.91-fold change), all of which have been previously implicated in the pathogenesis of arthritis [51–53]. The findings presented in this study indicate that PC is potentially an anti-inflammation agent. 

Interestingly, many genes classified in the inflammatory response were upregulated by PC, including adenosine A1 receptor ADORA1 (2.48-fold change), CD24 (2.09-fold change), and BCL6 (1.98-fold change). It is worth noting that previous studies demonstrated that the activation of adenosine receptors reduced inflammation and joint destruction in a rat adjuvant-induced arthritis, CD24 repressed tissue damage-induced immune response, and that BCL6 inhibited the expression of chemokines and attenuated allergic airway inflammation [54–56]. These previous findings indicate that ADORA1, CD24, and BCL6 are potentially anti-inflammation molecules. The upregulation of these genes by PC is consistent with the notion that PC is potentially an anti-inflammation agent [57]. 

The genes downregulated by PC included NFKBIZ (−2.58-fold change), ADRB2 (−2.41-fold change), and CXCL6 (−2.19-fold change). Interestingly, studies demonstrated that NFKBIZ mediated IL-6 production, ADRB2 antagonists reduced the severity of arthritis, and that anti-CXCL16 antibody inhibited infiltration of inflammatory cells and arthritis [58–60]. These previous findings indicate that NFKBIZ, ADRB2, and CXCL6 are proinflammation molecules. The downregulatory effect of PC on these genes is consistent with the notion that PC is potentially an anti-inflammation agent [57]. 

## 5. Conclusions

 These specific biological activities of PC are intrinsic properties of PC and are not dependent on the presence of calcium crystals. PC is potentially an anti-inflammation and meniscal protective agent. PC is not only potentially a disease-modifying drug for crystal-associated OA but also potentially a disease-modifying drug for arthritis associated with severe meniscal degeneration. The findings presented in this study provide further support for the development of PC, and/or its analogues, as disease-modifying drugs for OA therapy. 

## Figures and Tables

**Table 1:** Differentially expressed genes in PC-treated via untreated OA meniscal cells.

Biological process	Gene name	Gene ID	Differential expression*	Description
Cell proliferation	BLM	NM_000057	−7.41	Bloom syndrome, RecQ helicase-like
	NDP	NM_000266	−4.68	Norrie disease (pseudoglioma)
	HELLS	AF155827	−4.46	Helicase, lymphoid specific
	E2F7	AI341146	−4.12	E2F transcription factor 7
	CDC7	NM_003503	−4.11	Cell division cycle 7 homolog (*S. cerevisiae*)
	CDCA7	AY029179	−2.71	Cell division cycle-associated 7
	CDC25C	NM_001790	−2.36	Cell division cycle 25 homolog C (*S. pombe*)
	BRCA1	AF005068	−3.60	Breast cancer 1, early onset
	BRCA2	X95152	−3.30	Breast cancer 2, early onset
	PRKRA	AA279462	−3.34	PKinase, interferon-indu double-stranded RNA-dependent activator
	HHIP	AK098525	−3.28	Hedgehog interacting protein
	CHEK1	AA224205	−3.11	CHK1 checkpoint homolog (*S. pombe*)
	PTPRK	AU145587	−3.08	Protein tyrosine phosphatase, receptor type, *K *
	GINS1	NM_021067	−3.07	GINS complex subunit 1 (Psf1 homolog)
	TCF19	BC002493	−2.93	Transcription factor 19
	MKI67	AU147044	−2.92	Antigen identified by monoclonal antibody Ki-67
	PDS5B	AK026889	−2.88	PDS5, regulator of cohesion maintenance, homolog B
	UHRF1	AK025578	−2.77	Ubiquitin-like with PHD and ring finger domains 1
	AURKB	AB011446	−2.64	Aurora kinase B
	MKI67	AU132185	−2.64	Antigen identified by monoclonal antibody Ki-67
	FIGNL1	NM_022116	−2.62	Fidgetin-like 1
	KIF15	NM_020242	−2.60	Kinesin family member 15
	LRP6	NM_002336	−2.55	Low-density lipoprotein receptor-related protein 6
	ANXA1	AU155094	−2.47	Annexin A1
	DDX11	U33833	−2.47	DEAD/H (Asp-Glu-Ala-Asp/His) box polypeptide 11
	FANCA	AW083279	−2.46	Fanconi anemia, complementation group A
	SPRY2	NM_005842	−2.43	Sprouty homolog 2 (*Drosophila*)
	RECQL4	NM_004260	−2.43	RecQ protein-like 4
	NTN1	BF591483	−2.42	Netrin 1
	ADRB2	NM_000024	−2.41	Adrenergic, beta-2, receptor, surface
	CUL4A	AU155661	−2.40	Cullin 4A
	DLC1	NM_024767	−2.39	Deleted in liver cancer 1
	STIL	NM_003035	−2.39	SCL/TAL1 interrupting locus
	CHEK1	NM_001274	−2.37	CHK1 checkpoint homolog (*S. pombe*)
	TIMELESS	NM_003920	−2.37	Timeless homolog (*Drosophila*)
	SMAD4	AL832789	−2.23	SMAD family member 4
	SMAD1	NM_015583	−2.19	SMAD family member 1
	PCNA	NM_002592	−2.18	Proliferating cell nuclear antigen
	PRKCD	NM_006254	−2.17	Protein kinase C, delta
	RBBP4	AI972451	−2.16	Retinoblastoma binding protein 4
	PTGS1	NM_000962	−2.13	Prostaglandin-endoperoxide synthase 1
	ASPM	NM_018123	−2.13	Abnormal spindle (asp) homolog, microcephaly associated
	GLMN	AA814383	−2.11	Glomulin, FKBP associated protein
	NASP	NM_002482	−2.11	Nuclear autoantigenic sperm protein (histone binding)
	CCNA2	NM_001237	−2.09	Cyclin A2
	TBX2	AW173045	−2.08	T-box 2
	KIF2C	U63743	−2.08	Kinesin family member 2C
	PDCD1LG2	AF329193	−2.06	Programmed cell death 1 ligand 2
	BUB1B	NM_001211	−2.05	Budding uninhibited by benzimidazoles 1 homolog beta (yeast)
	POLA1	NM_016937	−2.04	Polymerase (DNA directed), alpha 1, catalytic subunit
	TACC3	NM_006342	−2.02	Transforming, acidic coiled-coil containing protein 3
	CDK2	M68520	−2.01	Cyclin-dependent kinase 2
	DLX5	NM_005221	2.99	Distal-less homeobox 5
	VCAM1	NM_001078	2.88	Vascular cell adhesion molecule 1
	ADORA1	NM_000674	2.48	Adenosine A1 receptor
	TNFRSF9	NM_001561	2.38	Tumor necrosis factor receptor superfamily, member 9
	FGF9	NM_002010	2.38	Fibroblast growth factor 9 (glia-activating factor)
	FABP3	NM_004102	2.24	Fatty acid binding protein 3, muscle, and heart
	FGFR2	M87771	2.20	Fibroblast growth factor receptor 2
	BAMBI	NM_012342	2.13	BMP and activin membrane-bound inhibitor homolog
	CD24	L33930	2.09	CD24 molecule
	HSF1	AI393937	2.07	Heat shock transcription factor 1

Ossification	COL13A1	M33653	−3.39	Collagen, type XIII, alpha 1
	SATB2	AK025127	−3.13	SATB homeobox 2
	ADRB2	NM_000024	−2.41	Adrenergic, beta-2, receptor, surface
	SMAD1	NM_015583	−2.19	SMAD family member 1
	ENPP1	BF591996	−2.02	Ectonucleotide pyrophosphatase/phosphodiesterase 1
	BMPR1B	AA935461	−1.93	Bone morphogenetic protein receptor, type IB
	NAB1	AF045452	−1.87	NGFI-A-binding protein 1 (EGR1-binding protein 1)
	GNAS	AA810695	−1.86	GNAS complex locus
	FGF18	BC006245	−1.73	Fibroblast growth factor 18
	TNFRSF11A	AW026379	−1.67	Tumor necrosis factor receptor superfamily, member 11a
	EGFR	K03193	−1.61	Epidermal growth factor receptor
	PLA2G4A	M68874	−1.61	Phospholipase A2, group IVA (cytosolic, calcium dependent)
	ANKH	T99215	−1.60	Ankylosis, progressive homolog (mouse)
	DLX5	NM_005221	2.99	Distal-less homeobox 5
	FGF9	NM_002010	2.38	Fibroblast growth factor 9 (glia-activating factor)
	IGFBP5	R73554	1.78	Insulin-like growth factor-binding protein 5
	GABBR1	N45228	1.72	Gamma-aminobutyric acid (GABA) B receptor, 1
	MMP14	NM_004995	1.72	Matrix metallopeptidase 14 (membrane inserted)
	TUFT1	NM_020127	1.66	Tuftelin 1
	COL1A1	AI743621	1.62	Collagen, type I, alpha 1
	MGP	NM_000900	1.61	Matrix Gla protein
	SMAD3	BF971416	1.60	SMAD family member 3

BMP signaling pathway	UBE2D3	AI239832	−2.44	Ubiquitin-conjugating enzyme E2D 3 (UBC4/5 homolog, yeast)
	ZFYVE16	BC032227	−2.43	Zinc finger, FYVE domain containing 16
	SMAD4	AL832789	−2.23	SMAD family member 4
	SMAD1	NM_015583	−2.19	SMAD family member 1
	BMPR1B	AA935461	−1.93	Bone morphogenetic protein receptor, type IB
	HIPK2	AW300045	−1.91	Homeodomain interacting protein kinase 2
	MSX1	AI421295	−1.84	Msh homeobox 1
	GREM2	NM_022469	−1.73	Gremlin 2, cysteine knot superfamily, homolog (Xenopus laevis)

Prostaglandin metabolic process	PTGS1	NM_000962	−2.13	Prostaglandin-endoperoxide synthase 1
	AKR1C2	BF508244	−1.91	Aldo-keto reductase family 1, member C2
	PLA2G4A	M68874	−1.61	Phospholipase A2, group IVA (cytosolic, calcium dependent)
	PTGS2	NM_000963	−*1.33 *	Prostaglandin-endoperoxide synthase 2
	PTGIS	D38145	1.79	Prostaglandin I2 (prostacyclin) synthase
	PDPN	AW590196	1.61	Podoplanin

Response to wounding	THBD	AW119113	−4.24	Thrombomodulin
	PLAT	NM_000930	−3.12	Plasminogen activator, tissue
	TFPI	J03225	−2.66	Tissue factor pathway inhibitor
	TFPI2	AL574096	−2.38	Tissue factor pathway inhibitor 2
	SDC1	NM_002997	−2.38	Syndecan 1
	SMAD1	NM_015583	−2.19	SMAD family member 1
	PLAUR	AY029180	−1.94	Plasminogen activator, urokinase receptor
	TMPRSS6	AI912086	1.96	Transmembrane protease, serine 6
	JUB	NM_032876	1.92	Jub, ajuba homolog (Xenopus laevis)

*Negative number indicates decreased expression and positive number indicates elevated expression (fold change) in PC-treated OA meniscal cells compared with untreated OA meniscal cells.

**Table 2:** Differentially expressed genes in PC-treated via untreated OA meniscal cells.

Biological process	Gene name	Gene ID	Differential expression*	Description
FGF receptor signaling pathway	FGF9	NM_002010	2.38	Fibroblast growth factor 9 (glia-activating factor)
	FGFR2	M87771	2.20	Fibroblast growth factor receptor 2
	FGF7	NM_002009	1.83	Fibroblast growth factor 7 (keratinocyte growth factor)
	NDST1	AL526632	1.71	N-deacetylase/N-sulfotransferase (heparan glucosaminyl) 1
	THBS1	AI812030	1.68	Thrombospondin 1
	HHIP	AK098525	−3.28	Hedgehog interacting protein
	FGF18	BC006245	−1.73	Fibroblast growth factor 18

Collagen fibril organization	COL14A1	M64108	2.06	Collagen, type XIV, alpha 1
	COL11A1	J04177	1.82	Collagen, type XI, alpha 1
	COL1A1	AI743621	1.62	Collagen, type I, alpha 1
	SERPINH1	NM_004353	1.66	Serpin peptidase inhibitor, clade H, member 1
	DPT	AI146848	1.65	Dermatopontin
	TRAM2	BC028121	1.60	Translocation-associated membrane protein 2
	COL5A1	AI983428	*1.52 *	Collagen, type V, alpha 1
	COL5A2	NM_000393	*1.44 *	Collagen, type V, alpha 2

Extracellular structure organization	CACNA1A	NM_023035	2.55	Calcium channel, voltage dependent, P/Q type, alpha 1A subunit
	COL14A1	M64108	2.06	Collagen, type XIV, alpha 1
	ECM2	NM_001393	2.04	Extracellular matrix protein 2, female organ, and adipocyte specific
	MYO6	AA877789	1.98	Myosin VI
	TMPRSS6	AI912086	1.96	Transmembrane protease, serine 6
	COL11A1	J04177	1.82	Collagen, type XI, alpha 1
	CRISPLD2	AL136861	1.74	Cysteine-rich secretory protein LCCL domain containing 2
	HSD17B12	BC012536	1.68	Hydroxysteroid (17-beta) dehydrogenase 12
	SERPINH1	NM_004353	1.66	Serpin peptidase inhibitor, clade H, member 1
	DCN	AI336924	1.65	Decorin
	DPT	AI146848	1.65	Dermatopontin
	COL1A1	AI743621	1.62	Collagen, type I, alpha 1
	APLP1	U48437	1.61	Amyloid beta (A4) precursor-like protein 1
	NFASC	AI821777	−1.99	Neurofascin homolog (chicken)
	MMP9	NM_004994	−1.91	Matrix metallopeptidase 9

Inflammatory response	ADORA1	NM_000674	2.48	Adenosine A1 receptor
	CD24	L33930	2.09	CD24 molecule
	BCL6	S67779	1.98	B-cell CLL/lymphoma 6
	NGF	NM_002506	1.84	Nerve growth factor (beta polypeptide)
	CFD	NM_001928	1.82	Complement factor D (adipsin)
	ITGB2	NM_000211	1.80	Integrin, beta 2
	CFI	BC020718	1.74	Complement factor I
	NDST1	AL526632	1.71	N-deacetylase/N-sulfotransferase (heparan glucosaminyl) 1
	IL17C	AF152099	1.70	Interleukin 17C
	THBS1	AI812030	1.68	Thrombospondin 1
	SERPINA3	NM_001085	1.67	Serpin peptidase inhibitor, clade A, member 3
	AGTR1	NM_004835	1.65	Angiotensin II receptor, type 1
	TFRC	N76327	1.64	Transferrin receptor (p90, CD71)
	PDPN	AW590196	1.61	Podoplanin
	MASP1	AI274095	1.61	Mannan-binding lectin serine peptidase 1
	NFKBIZ	BE646573	−2.58	NFKb inhibitor, zeta
	ANXA1	AU155094	−2.47	Annexin A1
	ADRB2	NM_000024	−2.41	Adrenergic, beta-2, receptor, surface
	SMAD1	NM_015583	−2.19	SMAD family member 1
	CXCL6	NM_002993	−2.19	Chemokine (C-X-C motif) ligand 6
	C2	NM_000063	−2.18	Complement component 2
	BMPR1B	AA935461	−1.93	Bone morphogenetic protein receptor, type IB
	TNFRSF1B	NM_001066	−1.74	Tumor necrosis factor receptor superfamily, member 1B
	EDNRA	NM_001957	−1.67	Endothelin receptor type A
	PLA2G4A	M68874	−1.61	Phospholipase A2, group IVA (cytosolic, calcium dependent)
	GPR68	AI805006	−1.60	G protein-coupled receptor 68

*Negative number indicates decreased expression and positive number indicates elevated expression (fold change) in PC-treated OA meniscal cells compared with untreated OA meniscal cells.

**Table 3:** Differential expression confirmed by real-time RT-PCR.

Gene name	Gene ID	Differential expression microarray	Differential expression* RT-PCR	Differential expression** RT-PCR
BLM	NM_000057	−7.41	−5.54	−6.54
HELLS	AF155827	−4.46	−4.17	−4.87
CDC25C	NM_001790	−2.36	−2.18	−2.92
CDC6	NM_001254	−2.00	−2.20	−3.41
CCNE2	NM_004702	−3.51	−2.94	−3.46
CCNA2	A1346350	−2.00	−2.34	−1.78
ANKH	T99215	−1.60	−1.42	−1.86
PTGS1	NM_000962	−2.13	−2.43	−2.98
THBD	AW119113	−4.24	−4.51	−3.79
FGF7	NM_002009	1.83	1.96	2.32
FGF9	NM_002010	2.38	2.47	2.70
COL11A1	J04177	1.82	1.96	2.01
ECM2	NM_001393	2.04	2.71	2.15
IGFBP5	R73554	1.78	1.95	2.34
CD24	L33930	2.09	2.32	3.10

*RT-PCR was performed using the RNA samples that were used for microarray analyses. **RT-PCR was performed using RNA samples from another experiment.
